# A Computational Model for Drug Release from PLGA Implant

**DOI:** 10.3390/ma11122416

**Published:** 2018-11-29

**Authors:** Miljan Milosevic, Dusica Stojanovic, Vladimir Simic, Bogdan Milicevic, Andjela Radisavljevic, Petar Uskokovic, Milos Kojic

**Affiliations:** 1 Bioengineering Research and Development Center BioIRC Kragujevac, Prvoslava Stojanovica 6, 34000 Kragujevac, Serbia; miljan.m@kg.ac.rs (M.M.); vladimir.simic.991@gmail.com (V.S.); dd11eeaann@gmail.com (B.M.); 2 Belgrade Metropolitan University, Tadeuša Košćuška 63, 11000 Belgrade, Serbia; 3 Faculty of Technology and Metallurgy, University of Belgrade, Karnegijeva 4, 11000 Belgrade, Serbia; duca@tmf.bg.ac.rs (D.S.); aradisavljevic@tmf.bg.ac.rs (A.R.); puskokovic@tmf.bg.ac.rs (P.U.); 4 Houston Methodist Research Institute, The Department of Nanomedicine, 6670 Bertner Ave., R7 117, Houston, TX 77030, USA; 5 Serbian Academy of Sciences and Arts, Knez Mihailova 35, 11000 Belgrade, Serbia

**Keywords:** computational modeling, radial finite element, composite smeared finite element, diffusion, emulsion electrospinning, controlled drug release

## Abstract

Due to the relative ease of producing nanofibers with a core–shell structure, emulsion electrospinning has been investigated intensively in making nanofibrous drug delivery systems for controlled and sustained release. Predictions of drug release rates from the poly (d,l-lactic-co-glycolic acid) (PLGA) produced via emulsion electrospinning can be a very difficult task due to the complexity of the system. A computational finite element methodology was used to calculate the diffusion mass transport of Rhodamine B (fluorescent drug model). Degradation effects and hydrophobicity (partitioning phenomenon) at the fiber/surrounding interface were included in the models. The results are validated by experiments where electrospun PLGA nanofiber mats with different contents were used. A new approach to three-dimensional (3D) modeling of nanofibers is presented in this work. The authors have introduced two original models for diffusive drug release from nanofibers to the 3D surrounding medium discretized by continuum 3D finite elements: (1) A model with simple radial one-dimensional (1D) finite elements, and (2) a model consisting of composite smeared finite elements (CSFEs). Numerical solutions, compared to experiments, demonstrate that both computational models provide accurate predictions of the diffusion process and can therefore serve as efficient tools for describing transport inside a polymer fiber network and drug release to the surrounding porous medium.

## 1. Introduction

The encapsulation and controllable release of drugs, as well as achieving enhanced therapeutic effects in drug delivery systems, were the subjects of investigation for a number of authors in the past (e.g., [1,2,3]). Among these drug-delivery systems, electrospun nanofiber mats are promising as drug carriers which offer site-specific delivery of drugs to the target in human body, and may be used for wound healing and cancer therapy [4,5,6,7,8]. Electrospinning is a technique that utilizes the electric force to drive the spinning process and to produce polymer fibers [9,10,11], and is capable of producing fibers with diameters in the nanometer range (10–1000 nm). Nanofibers obtained by electrospining are structurally homogeneous and are unlikely to encapsulate bioactive agents as nanoscaled particles. Recently, electrospinning of emulsions produced composite nanofibers with nanoscaled drug particles surrounded/coated by emulsifiers/surfactants and impregnated in biocompatible and/or biodegradable polymers [12]. Such types of composite nanofiber mats play the role of a controllable drug encapsulation/release vehicle.

The most commonly used biodegradable synthetic polymers for three-dimensional (3D) scaffolds in tissue engineering are saturated poly(α-hydroxy esters), including poly(lactic acid) (PLA) and poly(glycolic acid) (PGA), as well as poly(lactic-co-glycolide) (PLGA) copolymers [13,14]. PLGA has been well recognized for its suitability in drug delivery due to its good biocompatibility and ability to achieve complete drug release as a result of degradation and erosion of the polymer matrix. PLGA is a linear copolymer that can be prepared at different ratios between its constituent monomers, lactic (LA) and glycolic acid (GA). Depending on the ratio of lactide to glycolide used for the polymerization, different forms of PLGA can be obtained. The degradation of PLGA copolymer is the collective process of bulk diffusion, surface diffusion, bulk erosion, and surface erosion. Since there are many variables that influence the degradation process, the release rate pattern is often unpredictable. The release of a drug from the homogeneously degrading matrix is more complicated. Polymer composition is the most important factor for hydrophilicity and rate of degradation of a delivery matrix. Systematic studies of polymer composition with its degradation [15,16] showed that an increase in glycolic acid percentage accelerates the weight loss of polymer. It was shown that PLGA 50:50 (PLA/PGA) exhibited a faster degradation than PLGA 65:35 due to preferential degradation of glycolic acid proportion assigned by higher hydrophilicity. Subsequently, PLGA 65:35 shows faster degradation than PLGA 75:25, and so does PLGA 75:25 compared to PLGA 85:15 [17]. The absolute value of the degradation rate increases with the glycolic acid proportion. The amount of glycolic acid is a critical parameter in tuning the hydrophilicity of the matrix and, thus, the degradation and drug release rate [18].

Modeling the PLGA degradation and erosion is a prerequisite for drug release modeling, and mechanistic approaches are most commonly employed [19,20]. Accompanied and facilitated by PLGA degradation and erosion, drug release has a significant impact by changing the properties of the polymer matrix (porosity and PLGA MW), and such factors need to be captured in the diffusion drug transport models. The hydrophobicity of electrospun nanofiber mats could play an important role in the overall performances as tissue engineering scaffolds. While macromolecular hydrophilic drugs are limited by diffusion through the pore space, relatively smaller hydrophobic drugs could diffuse through both the PLGA matrix and the pore space [21].

It is a challenge to adequately model through numerical methods the process of drug release from fibers to the surrounding medium, with taking into account transport conditions within fibers (including degradation), in the medium, and at the interface between fibers and the surroundings. Using continuum elements for modeling fibers would require a huge effort for the finite element (FE) model generation and lead to an enormous number of equations, therefore preventing implementation to practical problems. In order to have a robust model, feasible for practical use, we have introduced two approaches here: (1) The use of a radial 1D finite element which replaces a detailed modeling of fibers by continuum elements [22], and (2) a model with fibers represented by a continuum according to the smeared concept introduced in References [23,24,25]. The second model is particularly attractive, since it does not need any 1D finite element meshing for fiber representation.

In the next section, Materials and Methods, electrospining methodology and the drug release of RhB from PLGA_1_ (65:35) and PLGA_2_ (50:50) nanofiber mats are investigated. Next, we present fundamental equations of the radial 1D element and the equation of degradation implemented into our model, including the hydrophobic effects. This is followed by the formulation of the smeared model for the fiber network. Finally, we demonstrate the applicability and accuracy of the computational models by presenting both numerical and experimental results.

## 2. Materials and Methods

Here are described the materials used for electrospining, as well as the electrospining procedure and the design of simple experiments for drug release.

### 2.1. Materials

Poly(d,l-lactide-co-glycolide) PLGA_1_ (average molecular weight MW 40,000–75,000 g/mol) with the mass ratio of lactide:glycolide units being (65:35), poly(d,l-lactide-co-glycolide) PLGA_2_ (average molecular weight MW 30,000–60,000 g/mol), with the mass ratio of lactide:glycolide units being (50:50), Rhodamine B (RhB), span-80, N,N-dimethylformamide (DMF) and chloroform (CHCl_3_), were purchased from Sigma-Aldrich Co. (Milwaukee, WI, USA). The chemicals were used without further purification. Phosphate buffered saline solution (PBS) was made by dissolving one tablet of PBS, supplied by Fisher Scientific (Hampton, NH, USA) in 200 mL of distilled water.

#### 2.1.1. Preparation of PLGA Nanofibers Produced via Emulsion Electrospinning

To prepare the emulsion containing 24 wt.% PLGA and 0.1 wt.% Rhodamine B, PLGA_1_ and PLGA_2_ (3.0 g) were initially dissolved in the mixture of solvents chloroform/DMF (8.25/2.75 g) and magnetically stirred at 200 rpm at room temperature for 24 h. After that, span-80 (50.0 mg) was added to this polymer solutions, followed by the addition of 5 wt.% of RhB aqueous solution (60 µL). The mixture was additionally stirred for 2 h.

**Figure 1:**
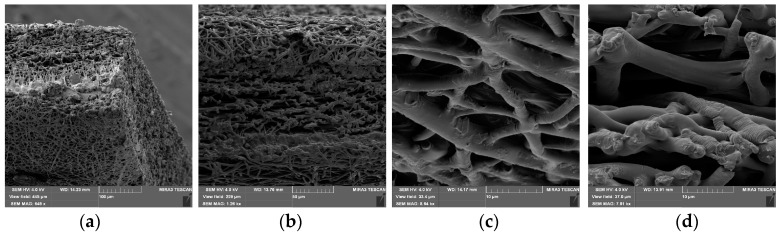
SEM images of drug loaded 24 wt.% 50:50 PLGA nanofibers (PLGA_2_).Images of same PLGA mat with different scale bars 100 µm (**a**), 50 µm (**b**), 10 µm (**c**) and 10 µm (**d**).

The vertical electrospinning experimental setup (CH-01, Linari Engineering, Pisa, Italy) was used as a method for the preparation of the nanofibers. In order to obtain fine nanofibers, the electrospinning conditions were optimized as follows: Polymer solutions were ejected from a 20 mL plastic syringe into a metallic needle (1 mm inner diameter) at a distance of 10 cm from collector, while flow rate was 3 mL/h and applied voltage 20 kV. SEM (scanning electron microscopy, Tescan Mira3 XMU (Brno, Czech Republic)) images of nanofibers are shown on Figure 1.

As shown in Figure 1a–d, the PLGA_2_ nanofiber scaffold is highly porous and has a dense mesh structure with bead-free and randomly arranged nanofibers. For the determination of the implant porosity, the nanofiber sheet’s apparent density was firstly estimated by the measurement of volume and mass of samples [26]. The results showed that for PLGA_1_ and PLGA_2_, the porosities were ~59% and ~78%, respectively. A numerical investigation regarding the influence of porosity was carried out in our previous paper. There, a radial 1D finite element for drug release from drug loaded nanofibers was introduced, where we confirmed that our model can predict the drug release for different porosities of the model [22].

#### 2.1.2. Drug Loading Efficiency

Rhodamine B encapsulation efficiency (EE) and the percentage of drug loading (DL) were determined using a UV spectrophotometer (UV Shimadzu 1700, Shimadzu Corporation, Kyoto, Japan) at 554 nm. The RhB encapsulation efficiency and the RhB loading capacity of the process were calculated according to the methods described in References [27,28]. Based on the obtained results, it was determined that the EE and DL for both PLGA_1_ and PLGA_2_ were 99% and 0.09%, respectively.

#### 2.1.3. In Vitro Drug Release Studies

The RhB-loaded PLGA_1_ and PLGA_2_ nanofiber mats were cut into small pieces, and approximately 40 mg (2.5 cm × 2.5 cm) of each sample was immersed in 20 mL phosphate buffer solution (PBS, pH = 7.4) at 37 °C. At certain time intervals, 1 mL of sample solution taken for the analysis was replaced with 1 mL of fresh PBS solution. This frequent process lasted for 2 months. The amount of RhB released in PBS at each time point was monitored by measuring the UV absorbance of the maximum peak for RhB (at an optical wavelength of 554 nm). The accumulated release of RhB was calculated based on a standard RhB absorbance-concentration calibration curve.

**Figure 2:**
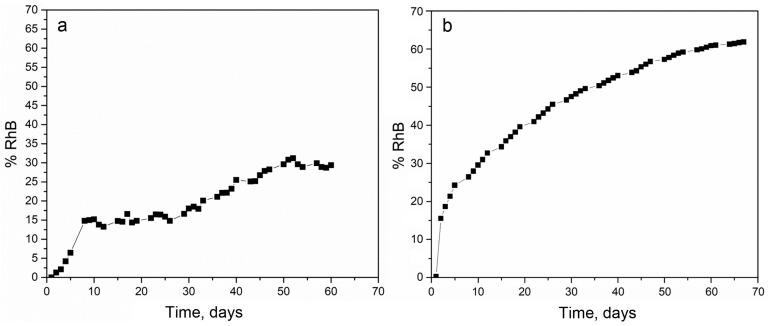
The experimental release of Rhodamine B (RhB) from poly(d,l-lactide-co-glycolide) PLGA_1_ (**a**) and PLGA_2_ (**b**) nanofiber mats.

The release of RhB from PLGA_1_ and PLGA_2_ nanofiber mats was investigated and is presented in Figure 2. Within the first 24 h, 0.3% was released from PLGA_2_, while release from PLGA_1_ was not observed. After two weeks, the total of ~34% of RhB was released from PLGA_2_ and ~14% from PLGA_1_. After 30 days, nearly 47% and 18% of the RhB was released from PLGA_2_ and PLGA_1_, respectively. At the end of the observed profile release period, PLGA_2_ nanofiber mat released ~60% and PLGA_1_ nanofiber mat released ~30% RhB. Based on the obtained release profiles, it can be concluded that PLGA_2_ has a faster release profile when compared to PLGA_1_. The release kinetics of incorporated RhB from PLGA electrospun nanofibers and the time required for hydrolytic degradation of PLGA depend on a molecular weight and chemical composition of polymers, porosity, crystallinity, hydrophobic/hydrophilic nature, as well as on a lactide/glycolide ratio. This is because low molecular weight PLGA generally leads to faster polymer degradation and a more rapid drug release. Additionally, as lactide is more hydrophobic than glycolide, an increase in lactide content in PLGA copolymers decreases the polymer degradation rate, followed by a slower drug release. The PLGA with 50:50 lactide to glycolide ratio had the fastest degradation rate (1–2 months), while the PLGA with 65:35 lactide to glycolide ratio degraded after 3–4 months.

## 3. Computational Models

In this section, we first summarize the basic equations for diffusion and degradation and then present formulation of the 1D and composite smeared finite element used to model diffusion within fibers.

### 3.1. Fundamental Equations

The diffusion domain model consists of fibers and surroundings filled with phenomenological fluid. The surroundings are modeled using 3D continuum elements, while fibers are approximated by radial 1D elements. The balance equation for diffusion in a 3D space, which is based on Fick’s law, can be written as:

(1) $$-\frac{\partial c}{\partial t}+\frac{\partial }{\partial x_{i}}\left(D_{ij}\frac{\partial c}{\partial x_{j}}\right)+q=0,\;\mathrm{sum}\;\mathrm{on}\;i,j;\;i=1,2,3,$$

where c is concentration, $D_{ij}$ are diffusion tensor coefficients, and q is a source term. In the case of 1D diffusion, this equation reduces to:

(2) $$-\frac{\partial c}{\partial t}+\frac{\partial }{\partial x}\left(D\frac{\partial c}{\partial x}\right)+q=0,$$

where D is diffusion coefficient for diffusion along the x-direction. This equation is used as the basic equation for diffusion within nanofibers.

Often, nanofibers are designed with a degradation and erosion process occurring during drug release. These effects can be taken into account by modifying the diffusion coefficient inside the fibers. We will include these effects in accordance with Reference [21]. Now, the diffusion coefficient of drug release through PLGA polymer is *D* = *D*(*M_w_*, *φ*), where *M_w_* is PLGA average molecular weight (MW) and *ϕ* is porosity. The function *D* = *D*(*M_w_*, *φ*) can be expressed as:

(3) $$D=\frac{(1-\phi )D_{s}+\kappa \phi D_{l}}{1-\phi +\kappa \phi },$$

where $D_{s}$ and $D_{l}$ are diffusivities of the polymer phase and liquid filled pores, respectively, and κ is partitioning (measure of hydrophobicity) between the liquid-filled pores and solid PLGA phase. Diffusivity in is given by the expression:

(4) $$D_{s}=D_{s0}{\left(\frac{M_{w}}{M_{w,0}}\right)}^{-\alpha },$$

where *D_s_*_0_ is diffusivity for the initial molecular weight *M*_*w*,0_ and *α* = 1.714 the experimentally determined coefficient. The molecular weight *M_w_* and porosity *ϕ* are functions of time *t*, described as:

(5) $$M_{w}=M_{w,0}\;e^{-k_{w}t},$$

and:

(6) $$\phi =\phi_{0}+(1-\phi_{0})(1+e^{-2kt}-2e^{-kt}),$$

where *k_w_* and *k* are degradation rate constants, taken as 2.5 × 10^−7^ s^−1^, and *φ*_0_(= 0) is the initial porosity.

### 3.2. Diffusion within Fibers

Two components of diffusion within a fiber can be distinguished: Axial, in the direction of the fiber axis, and radial, within the fiber cross-section.

#### 3.2.1. Axial Diffusion

This diffusion process is described by Equation (2) in differential form, which can be transformed into the finite element form by a standard Galerkin weighting procedure [29]. The FE balance equations can be written for a time step of size Δt and equilibrium iteration *i* as:

(7) $$\left(\frac{1}{\Delta t}M+K^{\left(i-1\right)}\right)\Delta C^{\left(i\right)}=\frac{1}{\Delta t}M\left(C^{\left(i-1\right)}-C^{t}\right)-K^{\left(i-1\right)}C^{\left(i-1\right)},$$

where matrices $M_{IJ}$ and $K_{IJ}$ are:

(8) $$M_{IJ}=\int_{L}N_{I}N_{J}AdL,\;K_{IJ}=\int_{L}DN_{I,x}N_{J,x}AdL,$$

where *N_I_*, *N_J_* are the interpolation functions, *A* is fiber cross-sectional area, and *L* is element length; **C** and **C**^*t*^ are nodal concentrations at the end and start of time step, respectively. Note that the balance equation, Equation (7), can be written for the continuum, using Equation (1), with matrices:

(9) $$M_{IJ}=\int_{V}N_{I}N_{J}dV,\;K_{IJ}=\int_{V}D_{ij}N_{I,i}N_{J,j}dV,$$

where V is the FE volume.

#### 3.2.2. Radial Diffusion

**Figure 3:**
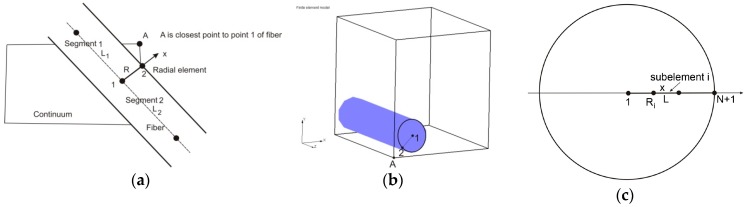
Definition of 1D radial finite element (**a**) and (**b**), and 1D radial element with subelements (**c**), according to Reference [22]. Copyright 2017. Reproduced with permission from the Journal of the Serbian Society for Computational Mechanics.

In Reference [22], a radial 1D finite element was formulated and we summarize the basic equations derived for this element here. A fiber is represented by a line composed of segments aligned on the fiber axis, with common points (Figure 3). At each common point, we generate one radial 1D element (fictitious in the FE mesh representation), which serves to represent radial diffusion within the fiber volume belonging to the common point. This belonging volume is equal to $\pi R^{2}\bar{L}$, where R is the mean radius of the length $\bar{L}$ belonging to the common point (it is (L_1_ + L_2_)/2 in Figure 3a).

With the above representation of the fibers, we are able to formulate a radial 1D finite element for the radial diffusion. At each common point of fiber segments, we introduce a radial 1D element which consists of two nodes, one in the middle of the fiber and another at the fiber surface. Hence, node 1 is at the symmetry axis of the fiber, while node 2 is at the fiber surface. As an approximation, it is considered that a node A of the 3D continuum (Figure 3b), closest to node 2 of the fiber, has the same concentration as node 2.

Now we can use the mass balance equations of the 2-node 1D FE element [23] as:

(10) $$\left(\frac{1}{\Delta t}M_{IJ}+K_{IJ}\right)\Delta C^{J}=-K_{IJ}C^{J}-\frac{1}{\Delta t}M_{IJ}\left(C^{J}-C^{Jt}\right),$$

where matrices $M_{IJ}$ and $K_{IJ}$ are:

(11) $$M_{IJ}=2\pi \bar{L}\int_{0}^{R}N_{I}N_{J}xdx,\;K_{IJ}=2\pi \bar{L}D_{fiber}\int_{0}^{R}N_{I,x}N_{J,x}xdx,$$

Other details are given in Reference [22]. The accuracy of the solution is increased if we use more than one radial element, as shown in Figure 3c, since the radial concentration profile is nonlinear. Then, the matrices for the radial subelements can be derived in analytical form as:

(12) $$M_{11}=2\pi \bar{L}\int_{0}^{L}\left(R_{i}+x\right)N_{1}N_{1}dx=2\pi \bar{L}L\left(\frac{1}{3}R_{i}+\frac{1}{12}L\right)\;M_{12}=2\pi \bar{L}\int_{0}^{L}\left(R_{i}+x\right)N_{1}N_{2}dx=2\pi \bar{L}L\left(\frac{1}{6}R_{i}+\frac{1}{12}L\right)\;M_{22}=2\pi \bar{L}\int_{0}^{L}\left(R_{i}+x\right)N_{2}N_{2}dx=2\pi \bar{L}L\left(\frac{1}{3}R_{i}+\frac{1}{4}L\right)$$

(13) $$K_{11}=K_{22}=-K_{12}=-K_{21}=\frac{2\pi \bar{L}}{L^{2}}D_{fiber}\int_{0}^{L}\left(R_{i}+x\right)dx=\frac{2\pi \bar{L}}{L}D_{fiber}\left(R_{i}+\frac{L}{2}\right),$$

where *R_i_* is the radius of node 1 of the current subelement (closer to the axis of symmetry) and *L* is the element length. The value of *D_fiber_* is the mean diffusivity for the element, calculated according to Equation (4) in cases when degradation and erosion are present. An expression for the initial concentration distribution in radial subelements is given in the Appendix A.

### 3.3. Fundamental Equations for CSFE

Here, we summarize the methodology which will be further used as the basis for the development of the composite smeared finite element to model diffusion within a fiber network and the surrounding. It has the analogy with our representation of mass transport within a capillary system and surrounding tissue [23].

**Figure 4:**
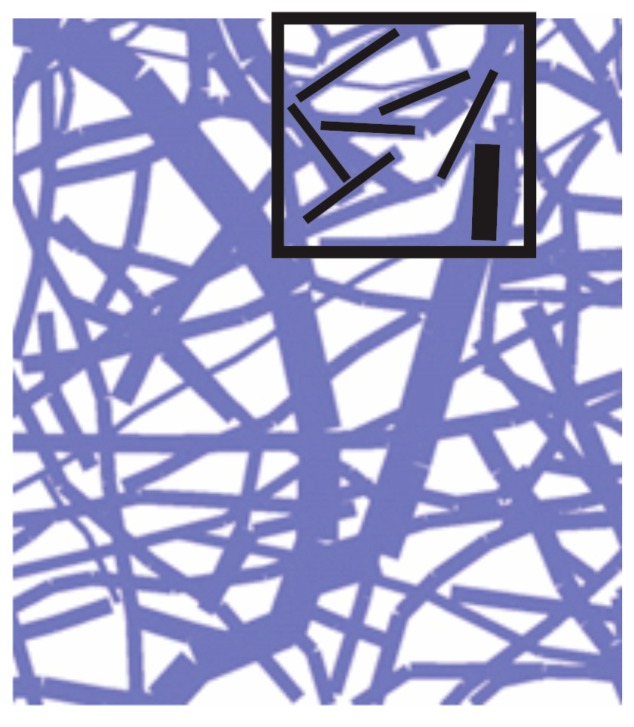
Network of fibers and a domain of finite element with indicated fiber directions (domain is 90 μm × 90 μm).

First, considering the axial diffusion in fibers, we transform the 1D diffusion along the fibers directions to the corresponding equivalent continuum form. This is achieved by derivation of the continuum diffusion tensor, which can be expressed in the following equation [23]:

(14) $$D_{ij}=\frac{1}{A_{tot}}\sum_{K}D_{K}A_{K}{𝓁}_{Ki}{𝓁}_{Kj},$$

where summation includes all fibers in the neighborhood of a considered point (or within a finite element), $D_{K}$ and $A_{K}$ are diffusion coefficients an cross-sectional areas, ${𝓁}_{Ki}$ are directional cosines, and $A_{tot}$ is the total fiber cross-sectional area (sum of $A_{K}$). Fibers and finite element are schematically shown in Figure 4. Therefore, instead of using Equation (10) for each fiber, we can use continuum elements with the matrices given in Equation (9), with diffusion coefficients evaluated according to Equation (14).

**Figure 5:**
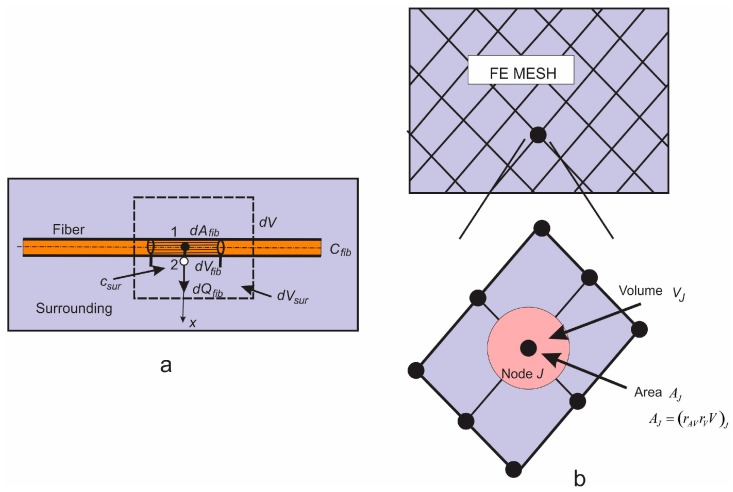
(**a**) Diffusion from fiber surface *dA_fib_*_,_ which corresponds to the fiber volume *dV_fib_* and total volume *dV*; *dV_sur_* is the volume occupied by the surrounding medium; (**b**) Corresponding smeared model.

Next, radial diffusion in the smeared concept can be formulated as follows. Consider diffusion through a fiber as schematically shown in Figure 5. First, the elementary area of the surface of the fiber wall $dA_{fib}$ can be related to the elementary volume $dV_{fib}$ and further to the elementary total volume dV, as follows:

(15) $$dA_{fib}=r_{AV}dV_{fib}=r_{AV}r_{V}dV,$$

where $r_{AV}$ is the fiber area-to-volume ratio (called further surface ratio) and $r_{V}$ is the fibers’ volumetric ratio within the surrounding medium, or fiber density; the volume of surrounding is $\left(1-r_{V}\right)dV$. Note that in the case of a straight fiber, the surface ratio is $r_{AV}=4/D_{fib}$, where $D_{fib}$ is the fiber diameter. Equation (15) can be considered the most fundamental in our smeared models, where the discrete fiber surface is smeared over the volume of the continuum.

Next, we assume that the mass concentration is linearly distributed along the fiber radius (between points 1 and 2 in Figure 5a). Then, the flux from the fiber at point 2, corresponding to the elementary surface $dA_{fib}$, including partitioning P at the fiber surface, can be expressed as:

(16) $$dQ_{fib}=\left[D\left(C_{fib}-PC_{sur}\right)-\frac{R}{6\Delta t}{\left(C-C^{t}\right)}_{fib}-\frac{R}{3\Delta t}{\left(PC-C^{t}\right)}_{sur}\right]r_{AV}r_{V}dV,$$

where $C_{fib}$, $C_{fib}^{t}$, $C_{sur}$, and $C_{sur}^{t}$ are the fiber and surrounding concentrations at the end and start of time step, respectively, and R is radius of the fiber. Note that D represents the overall transport coefficient through the fiber (with degradation and erosion). Therefore, considering the surrounding medium, we have distributed mass source terms, according to Equation (16), which can be associated with the integration points of the 3D finite elements. The nodal fluxes of a continuum finite element are:

(17) $$Q_{fibI}=\int_{V}N_{I}dQ_{fib}=\int_{V}N_{I}\left(\ldots \right)\left(1-r_{V}\right)dV,$$

where terms within the parenthesis (…) follow from Equation (16), and $N_{I}$ is the continuum interpolation functions of the element with the volume V. When evaluating the integral Equation (17), $C_{fib}$ and $C_{sur}$ are interpolated (from FE nodes) concentrations at the element integration point of fibers and within the surrounding. Note that the factor $\left(1-r_{V}\right)$ is used, since the volume of tissue is reduced due to the presence of fibers.

Instead of using source terms at FE integration points, connectivity elements can be introduced and assigned at each continuum node. Then, the balance equation for the connectivity element at a continuum node *I* can be expressed in Equation (10), where $C_{1}=C_{fib}$, $C_{2}=C_{sur}$ at the node *I*, and the matrices are:

(18) $$M_{11}=\frac{1}{3}A_{fibI}R_{I},\;M_{12}=M_{21}=\frac{1}{6}P_{I}A_{fibI}R_{I},\;M_{22}=P_{I}M_{11}\;K_{11}=A_{fib}D_{fibI},\;K_{22}=-K_{12}=-K_{21}=P_{I}\;K_{11},$$

where (at node *I*) $P_{I}$ is partitioning coefficient as in Equation (16); $D_{fibI}$ is the fiber diffusion coefficient; $R_{I}$ is the fiber radius; and $A_{capI}$ is the fiber surface area belonging to the node *I*, which is:

(19) $$A_{fibI}={\left(r_{AV}r_{V}\right)}_{I}V_{I},$$

with ${\left(r_{V}\right)}_{I}$, ${\left(r_{AV}\right)}_{I}$ being the volumetric ratio and the area coefficient; and $V_{I}$ being the volume of the continuum, which belongs to the node (schematically shown in Figure 5b). The volume $V_{I}$ can be numerically evaluated as:

(20) $$V_{I}=\sum_{elements}\int_{V}N_{I}dV,$$

where summation includes all elements containing node *I*. It is important to note that nodes representing one connectivity element have the same spatial position. We found that convergence was improved by applying the concept of these connectivity elements instead of continuously distributed source terms given by Equation (17).

It can be concluded from the above that diffusive transport between fibers and tissue can be performed by discretizing the continuum only. The parameters of the model, assigned to each continuum node *I*, include geometrical data (the volumetric ratio of fibers) ${\left(r_{V}\right)}_{I}$, the surface ratio ${\left(r_{AV}\right)}_{I}$, mean radius of fibers ($R_{I}$), and material data of fibers consisting of diffusion coefficient $D_{I}$ and partition coefficient $P_{I}$ at the fiber surfaces.

**Figure 6:**
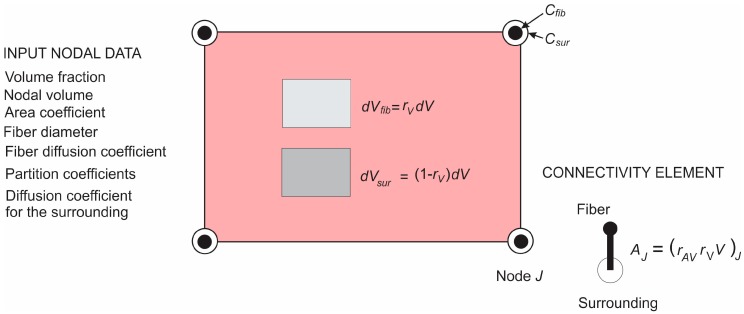
Composite smeared finite element (CSFE) with fiber and surrounding domain and connectivity elements at each node; a list of nodal parameters is given in the figure.

Finally, we describe the composite smeared finite element, which includes the fiber domain and the surrounding domain, coupled by the connectivity elements at each FE node. A schematic of this element is shown in Figure 6. The volume V of the element is occupied by the fiber domain $r_{V}V$ and by the surrounding medium $(1-r_{V})V$. The model parameters are also given in the figure.

## 4. Numerical and Experimental Results of Drug Release

**Figure 7:**
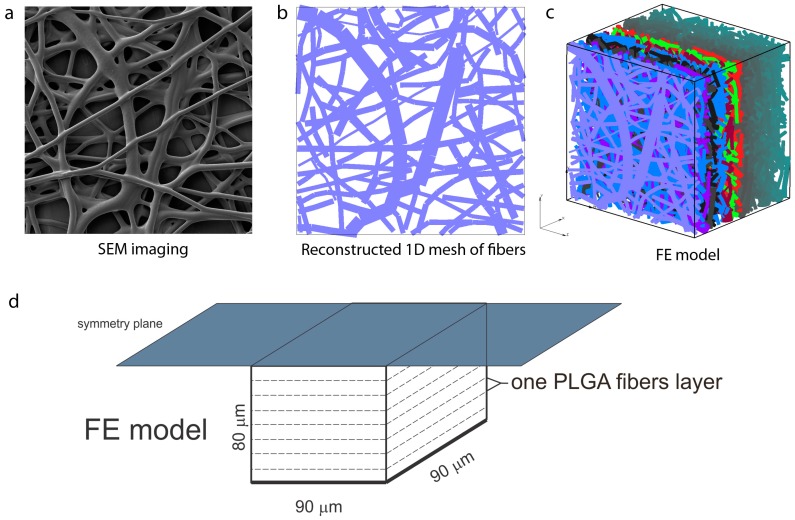
Configuration of the finite element (FE) model of PLGA implant, (**a**) SEM imaging of one layer of PLFA fibers, (**b**) reconstructed 1D mesh of fibers (scan bar 20 μm), (**c**) reconstructed FE model, (**d**) configuration and geometry of FE model [22]. Copyright 2017. Reproduced with permission from the Journal of the Serbian Society for Computational Mechanics.

As mentioned in Section 2, a complete PLGA implant is with dimensions of 2.5 cm × 2.5 cm, with thickness of 160 µm. The morphology of the nanofiber mats was examined by electron microscopy (SEM) scanning, performed at the Faculty of Technology and Metallurgy, Figure 7. The network of fibers is reconstructed from an SEM image of 90 µm × 90 µm using indoor software, Figure 7b. Two different models are generated: (a) A detailed FE model with 1D radial elements and (b) a composite smeared finite element model with two different domains: Fiber domain and surrounding domain. Both numerical models are built in our FE program PAK (in Serbian: Program za Analizu Konstrukcija—Program for Structural Alalysis) [30]. Conclusions regarding differences in the hydrophobicity and degradation of PLGA_1_ and PLGA_2_ are taken from References [12,13,14,15,16,17,18]. We adopted the following conditions in our FE models:

- Hydrophobicity (partitioning) of drug transport within PLGA_1_ is lower than for PLGA_2_;
- Degradation of PLGA_1_ is much slower than degradation of PLGA_2_.

### 4.1. Preparation and Numerical Simulation of Detailed FE Models

By randomly duplicating and displacing the generated layer of 1D fibers into the longitudinal direction of the modeling domain, we can generate a mat of fibers within any implant (Figure 7c,d). Further, assuming symmetric conditions, we can model just one half of the implant. It is also reasonable to adopt a homogenous distribution (or repetition) of one small domain of the fibers, through which we can model just one part of the implant. Thus, the dimensions of our FE models are: 80 µm × 90 µm × 90 µm. By detailed analysis (not shown here), we found that under the conditions considered for this implant, diffusion from the fibers is dominantly radial, and we consequently included radial elements only in our model. The 3D FE mesh is composed of 64,512 nodes and 36,864 elements; the number of radial 1D elements is around 7580 for the considered examples. Diffusion transport within the 3D FE mesh of the surrounding is affected by the position and orientation of fibers. Since 3D mesh and 1D mesh are independent, it is assumed that the material point of the 3D domain which is geometrically within the fiber is not used in the FE calculation. That point is considered the so-called “immersed” point.

We assume that the diffusion coefficient of Span 80/RhB in pore space (space between fibers) is as in water, and the diffusion coefficient of Span 80/RhB within the fibers (fiber with impregnated drug inside) is *D_fiber_* = 4 × 10^−10^ cm^2^/s, which is taken from Reference [31], approximately 104 times lower than in water. The time period of simulation was 75 days (15 time steps with 5 days each). The boundary conditions of the model are: Prescribed concentration C_0_ in fibers and C = 0 at outer boundary of implant (boundary where mass release is measured).

The model used for Span 80/RhB diffusion is shown in Figure 7. The parameters of the detailed model are:

- Dimensions: 80 µm × 90 µm × 90 µm;
- FE mesh: 40 × 48 × 48 divisions;
- Diffusion coefficient within fibers: *D_fiber_* = 0.04 µm^2^/s;
- Diffusion coefficient in between fibers: *D_liquid_* = 0.04 µm^2^/s;
- Mean diameter: *D* = 2.5 µm.

### 4.2. Application of Smeared Modeling for Drug Transport in PLGA Implant

Using our recently introduced composite smeared finite element concept [23], we were able to model the transport process of drug transport from fibers to borders of implant. The smeared model consists of two domains:

- Fiber domain—equivalent domain of fibers;
- Surrounding domain—equivalent “pore” space surrounding fibers.

The input parameters of the model are:

- Volume fraction of fibers in PLGA layers;
- Diffusion coefficient within PLGA fiber, for either 24 wt.% 50:50 and 65:35 emulsion;
- Diffusion coefficient of drug within the surrounding domain. Coefficient of hydrophobicity (partitioning);
- Mean diameter of PLGA fibers.

**Figure 8:**
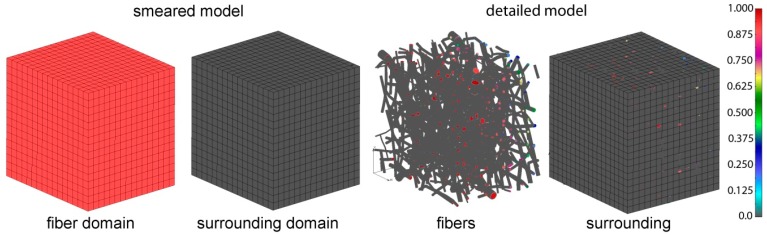
PLGA domain modeled using a smeared composite finite element or detailed model with the mesh of fibers.

The detailed and corresponding smeared model we used for drug transport analysis of 24 wt.% 50:50 and 65:35 emulsion are shown on Figure 8.

In order to determine the diffusion coefficient for the surrounding domain, a numerical homogenization procedure was performed according to Reference [32]. It was found that diffusion is reduced in the surrounding due to the attractive forces between fibers and diffusion molecules, and also by the presence of fibers in the system. The equivalent diffusion coefficient in the surrounding domain was found to be *D_liquid_* = 0.004 µm^2^/s. The parameters used in our composite smeared finite element (CSFE) model are:

- Volume fraction of fibers: $r_{V}$= 0.4223;
- Mean diameter of fibers: *D* = 2.5 µm;
- Diffusion coefficient within fibers: *D_wall_* = 0.04 µm^2^/s;
- Equivalent diffusion coefficient in surrounding domain: *D_liquid_* = 0.004 µm^2^/s;
- Partitioning: *P* = 1.

### 4.3. Comparation of Numerical and Experimental Results

**Figure 9:**
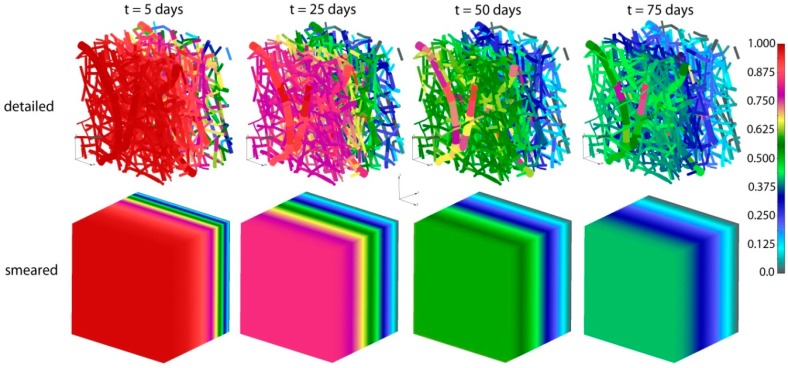
PLGA implant—concentration field for the detailed and smeared model, for the diffusion of Span-80/RhB complex within the PLGA implant.

**Figure 10:**
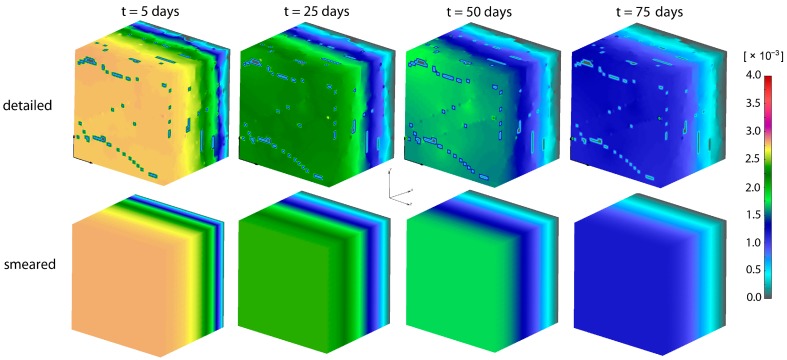
PLGA implant—concentration filed in the surrounding domain, for the detailed and smeared model, for the diffusion of Span-80/RhB complex within the PLGA implant.

Concentrations for both detailed and smeared models of PLGA_1_ are shown in Figure 9 and Figure 10, for domains with fibers and the surrounding domain, in a period of 75 days. It can be seen that there are small differences between the two models; hence, the smeared modeling concept can be used for the prediction of drug transport from drug impregnated nanofibers.

**Figure 11:**
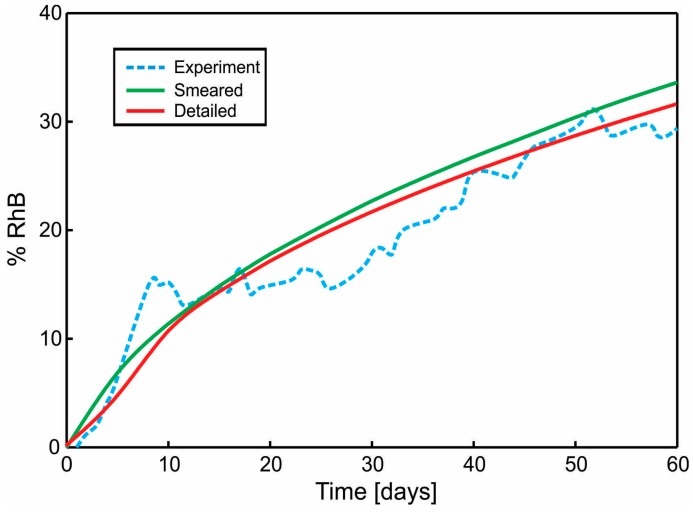
Cumulative Release vs. Time for Span-80/RhB complex impregnated and for 24 wt.% 65:35 PLGA. Experimental curve and computational results obtained using the true (detailed) and smeared model of PLGA nanofibers.

**Figure 12:**
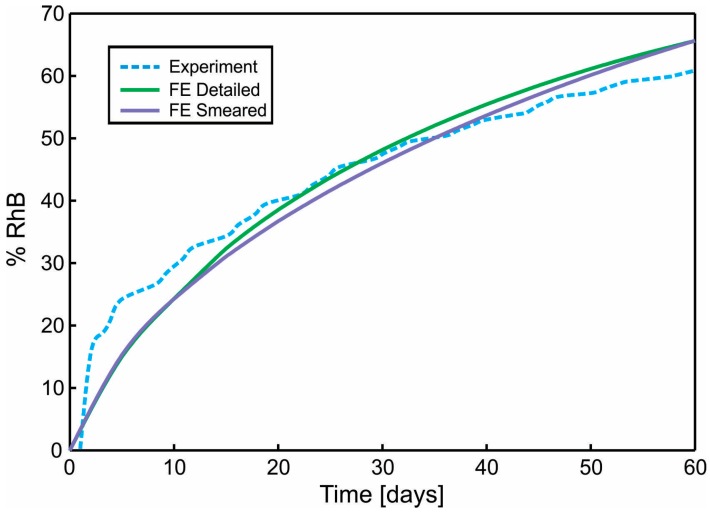
Diagram Cumulative Release vs. Time. Comparison of results for the true (detailed) and smeared model of PLGA nanofibers, with Span-80/RhB complex impregnated and for 24 wt.% 50:50 PLGA. Experimental curve and computational results obtained using true (detailed) and smeared model of PLGA nanofibers.

A diagram of cumulative mass release obtained from an experiment conducted at the Faculty of Technology and Metalurgy and an FE simulation of PLGA_1_ and PLGA_2_ (both detailed and smeared model) are given in Figure 11 and Figure 12. In a numerical simulation, for PLGA_1_, we used partitioning coefficient *P* = 2 × 10^5^ and degradation coefficient $\kappa_{w}$ = 2.5 × 10^−7^ s^−1^, while for PLGA_2_, the partitioning coefficient was *P* = 5 × 10^5^ and degradation coefficient $\kappa_{w}$ = 2.0 × 10^−7^ s^−1^. Coefficients used in simulations are in accordance with experimental studies, where it is stated that PLGA 50:50 (PLA/PGA) exhibits a faster degradation than PLGA 65:35, and higher hydrophilicity (which means a smaller partitioning coefficient).

## 5. Discussion

The use of implants for drug delivery according to a desirable rate and over a long time period is a promising approach in modern medicine. One attractive concept is to design implants composed of drug loaded nanofibers immersed into the appropriate surrounding medium. There are technological challenges with respect to electrospinning in producing biodegradable nanofibers with the needed characteristics. Our experimental approach was presented in this work.

Computational models would be a very useful tool, which can help in implant drug delivery systems overall. Since the process of drug release from nanofibers and implants is very complex, the development of appropriate computational models which can capture this complexity also represents a challenge. A most straightforward approach would be to generate a 3D finite element model which, in detail, follows an irregular fiber network and the surrounding. It is not only very demanding to generate such complex FE mesh, but the model will be huge with respect to number of equations to be solved over time. To make a model feasible for practical applications, we introduced 1D (line segments) representation of fibers with the axial and radial diffusion. The radial diffusion is modeled by specific 1D radial elements [22], summarized here, which connect diffusion from the fiber axis of line elements and the 3D finite element model of the surrounding. This model, called a detailed model, is computationally efficient, but still requires a detailed 1D mesh of the fiber network. The most important novelty of this work is that we have formulated a smeared FE model which is analogous to our smeared model for mass transport from a capillary network to the biological tissue [23]. The 1D fiber axial diffusion is substituted by a 3D continuum representation with the appropriately derived diffusion tensor for continuum. The radial diffusion is captured by connectivity elements at each FE node, which connect concentrations within fibers and the surrounding and include partitioning at the surface of fibers. The required geometrical data here consist only of the fiber volumetric fraction and diameters at the FE nodes.

The selected examples showed that both detailed and smeared models give results which match well with experimental findings. We found that radial diffusion from fibers is dominant.

Finally, we note that the presented methodology is applicable to other porosities, drugs and diffusion coefficients, rate of degradation, and hydrophobicity (here, we used data for PLGA_1_). The accuracy of our models is already shown in our references, for different material data sets [23,24,25].

## 6. Conclusions

New computational modeling approaches using FEM are developed in order to simulate drug transport from drug loaded nanofibers. Those approaches incorporate partitioning and degradation effects, which are present and may dominate in drug delivery from commonly fabricated nanofibers employed in the design of implants.

Both detailed and smeared modeling concepts offer an accurate prediction of drug release from nanofibers. However, the computational model with smeared finite elements for axial drug diffusion and connectivity elements for the radial diffusion, presented as a novel concept in in this work, offers an efficient tool, which is accurate and simple for application. This model is particularly attractive for drug transport within multilayered nanoimplants used in tissue engineering, cancer healing, and postoperative therapy.

## Appendix A. Initial Concentrations for Radial Subelements

Here, we provide the equation for the determination of initial drug concentration at fiber nodes in cases when the number of subelements is greater than 2, for the detailed FE model with 1D radial finite elements [26]. The number of subelements affects the value of initial concentration that has to be prescribed at fiber nodes at the start of FE simulation. The mass of drug within the fiber segment is:

(A.1) $$m=2\pi \bar{L}c_{0}\int_{R}x\cdot dx=R^{2}\pi \bar{L}c_{0},$$

where $c_{0}$ is initial concentration of the impregnated drug. In a case with n subelements, the concentration within first *n* − 1 elements can be considered constant, while in the last subelement, it is in a range from $c_{0}^{n}$ to 0, where $c_{0}^{n}$ is the initial concentration which has to be determined. In such cases, we determine that mass within a fiber segment, consisting of n subelements, is:

(A.2) $$m_{n}=m_{n1}+m_{n2}=2\pi \bar{L}c_{0}^{n}\int_{0}^{R_{n-1}}xdx+2\pi \bar{L}\int_{R_{n-1}}^{R}c_{x}xdx,$$

where $m_{n1}$ is mass within first *n* − 1 segments, $m_{n2}$ is mass in the last nth segment, and:

(A.3) $$R_{n-1}=\frac{n-1}{n}R,$$

is the radius at the first node of the nth subelement. Concentration along the nth sub-element is linearly changing according to the following equation:

(A.4) $$c_{x}=(1-\frac{x-R_{n-1}}{R-R_{n-1}})c_{0}^{n},$$

Using Equation (A.3), it follows from Equation (A.4):

(A.5) $$c_{x}=c_{0}^{n}\frac{n}{R}(R-x),$$

where x is changing from $R_{n-1}$ to R, with $R-R_{n-1}=R/n$. Now we have the following equations:

(A.6) $$m_{n1}={\left(\frac{n-1}{n}\right)}^{2}R^{2}\pi Lc_{0}^{n}\;m_{n2}=2\pi \bar{L}c_{0}^{n}\frac{n}{R}\int_{\frac{n-1}{n}R}^{R}\left(R\cdot x\cdot dx-x^{2}\cdot dx\right)=\left(\frac{3n-2}{3n^{2}}\right)R^{2}\pi \bar{L}c_{0}^{n},$$

from which it follows:

(A.7) $$m_{n}=m_{n1}+m_{n2}=\left(\frac{3n^{2}-3n+1}{3n^{2}}\right)R^{2}\pi \bar{L}c_{0}^{n}.$$

Using Equations (A.1) and (A.7), we have, finally:

(A.8) $$c_{0}^{n}=\frac{3n^{2}}{3n^{2}-3n+1}c_{0}.$$

This is the value that has to be prescribed at each node of subelements as the initial concentration, except at the node on the fiber surface, which is equal to zero.
